# Pharmacological Analysis of P2Y Purinergic Receptors That Modulate the Rat Vasopressor Sympathetic Tone

**DOI:** 10.3390/ph19101594

**Published:** 2026-10-08

**Authors:** Carlos M. Villalón, Juan Linares-Bedolla, Belinda Villanueva-Castillo, Alejandro D. Miguel-Martínez, Kristian A. Haanes, Antoinette MaassenVanDenBrink, Juan F. Fernández-González, Eduardo Rivera-Mancilla

**Affiliations:** 1 Departamento de Farmacobiología, Cinvestav-Coapa, Czda. de los Tenorios 235, Col. Granjas Coapa, Deleg. Tlalpan, Ciudad de Mexico C.P. 14330, Mexico; juan.linares@cinvestav.mx (J.L.-B.); belyndavc@hotmail.com (B.V.-C.); dralejandrodanielmmtz@gmail.com (A.D.M.-M.); 2 Translational Research Centre, Copenhagen University Hospital—Rigshospitalet, Nordstjernevej 42, 2600 Glostrup, Denmark; kristian.agmund.haanes@regionh.dk; 3 Department of Health Technology, Technical University of Denmark, 2800 Kongens Lyngby, Denmark; 4 Danish Headache Center, Department of Neurology, Copenhagen University Hospital—Rigshospitalet, 2600 Glostrup, Denmark; 5 Division of Vascular Medicine and Pharmacology, Erasmus MC University Medical Center Rotterdam, P.O. Box 2040, 3000 CA Rotterdam, The Netherlands; a.vanharen-maassenvandenbrink@erasmusmc.nl (A.M.); e.riveramancilla@erasmusmc.nl (E.R.-M.); 6 Laboratorio de Farmacología, Departamento de Fisiología y Farmacología, Facultad de Farmacia, Universidad de Salamanca, Campus Miguel de Unamuno, C. Lic. Méndez Nieto, s/n, 37007 Salamanca, Spain; juanfdezglez@usal.es

**Keywords:** ADPβS, glibenclamide, pithed rat, P2Y_1_ receptor, P2Y_12_ receptor, P2Y_13_ receptor, K_ATP_ channels, sympathoinhibition, neurotransmission, cardiovascular pharmacology

## Abstract

**Background/Objectives:** Adenosine 5′-O-(β-thio)-diphosphate (ADPβS) is a stable analogue of adenosine diphosphate (ADP). In pithed rats, ADPβS produces (i) inhibition of both the cardioaccelerator sympathetic tone (via prejunctional P2Y_12_/P2Y_13_ receptors) and the vasodepressor sensory CGRPergic tone (via prejunctional P2Y_1_ receptors) and (ii) vasodepressor/vasodilator responses via endothelial P2Y_1_ receptors, and vasopressor/vasoconstrictor responses via P2Y_1_/P2Y_12_/P2Y_13_ receptors. This study pharmacologically investigates whether P2Y_1_, P2Y_12_, and/or P2Y_13_ receptors modulate the vasopressor sympathetic tone using ADPβS. **Methods:** A total of 126 male Wistar rats were pithed and classified into two groups to produce vasopressor responses by (i) spinal (T_7_–T_9_) electrical stimulation of the sympathetic vasopressor tone (0.03–3 Hz; *n* = 102) and (ii) intravenous (i.v.) boluses of noradrenaline (0.03–3 μg/kg; *n* = 24). **Results:** Continuous i.v. infusions of ADPβS (5.6 and 10 μg/kg·min) significantly inhibited the vasopressor responses to sympathetic electrical stimulation, without affecting those induced by exogenous noradrenaline. This sympathoinhibition by ADPβS (5.6 μg/kg·min) remained unchanged after vehicle (1 mL/kg, i.v.) or the antagonists PSB 0739 (P2Y_12;_ 300 μg/kg, i.v.) and MRS 2211 (P2Y_13;_ 1000 and 3000 μg/kg, i.v.). Conversely, it was abolished after MRS 2500 (P2Y_1;_ 300 μg/kg, i.v.) or glibenclamide (K_ATP_ channel blocker; 20 mg/kg, i.v.). **Conclusions:** ADPβS inhibits the vasopressor sympathetic tone via prejunctional P2Y_1_ receptors through K_ATP_ channel-dependent mechanisms.

## 1. Introduction

Purinergic signalling modulates cardiovascular function by integrating receptor activity across both central and peripheral pathways [1,2,3]. Consequently, dysregulation of this signalling can alter neurogenic vascular and cardiac control, leading to various cardiovascular disorders, including hypertension [1,4,5]. These integrated pathways encompass the central nervous system (CNS), preganglionic neurons, autonomic ganglia, the adrenal medulla, postganglionic neurons, peptidergic sensory fibres, blood vessels (including the endothelium), and the heart [1,2,3,4,5,6,7].

This purinergic system is triggered by extracellular nucleotides such as adenosine triphosphate (ATP), which can be hydrolysed to adenosine diphosphate (ADP) by the action of ectonucleotidases and subsequently into smaller molecules, including adenosine [8]. These resulting purine derivatives can activate three main families of purinergic receptors classified as follows:

(i) **P1 receptors**, which are stimulated by adenosine and have been subclassified into the A_1_ (G_i_-coupled), A_2A_ (G_s_-coupled), A_2B_ (G_s_-coupled), and A_3_ (G_i_-coupled) subtypes [9,10]. These receptors modulate cardiovascular and neuronal function [11].
(ii) **P2Y receptors**, which have been subclassified (in mammals) into the P2Y_1_, P2Y_2_, P2Y_4_, P2Y_6_, P2Y_11_, P2Y_12_, P2Y_13_, and P2Y_14_ subtypes and can be stimulated by ATP, ADP, UTP, UDP or UDP-glucose, depending on the specific subtype involved [9,12,13,14,15]. It is unclear whether the P2Y_5_, P2Y_7_, P2Y_9_, and P2Y_10_ subtypes are nucleotide receptors [15,16]. Transductionally, the P2Y_1_, P2Y_2_, P2Y_4_, P2Y_6_ and P2Y_11_ subtypes operate through G_q_-mediated signalling, whereas the P2Y_11_ subtype also involves G_s_-mediated signalling [7,9,12,13,14]; in contrast, the P2Y_12_, P2Y_13_, and P2Y_14_ subtypes engage G_i_-mediated signalling [7,9,12,13,14]. Crucially, seminal in vitro data have reinforced this cellular background by demonstrating that complex transductional signalling pathways and differential G_q_/G_i_-protein interactions within vascular tissues critically orchestrate downstream responses [17]. These receptors modulate, among others, platelet function, autonomic/sensory neurotransmission, endothelial signalling, and vascular tone [17,18,19].
(iii) **P2X receptors**, which are an integral part of cation channels activated by ATP and consist of seven subtypes (P2X_1_–P2X_7_). These receptors participate in rapid excitatory responses in immune cells, neurons, vascular smooth muscle, fast synaptic transmission, and neurogenic vascular modulation [1,2,3,5,6].

On this basis, ATP can mediate fast excitatory transmission by ionotropic P2X receptors, whereas its extracellular degradation generates ADP, which preferentially activates metabotropic P2Y receptors and exerts slower, modulatory effects. Due to the susceptibility of ADP to degradation by ectonucleotidases [8], many studies have used the stable and non-hydrolysable ADP analogue adenosine 5′-O-(β-thio)-diphosphate (ADPβS), which is fundamental for investigating purinergic signalling involvement in cardiovascular modulation. Indeed, ADPβS is an agonist that preferentially stimulates P2Y_1_, P2Y_12_ and P2Y_13_ receptors [1,2,3,12,20].

Furthermore, intravenous (i.v.) doses of ADPβS cause reductions in diastolic blood pressure (i.e., systemic vasodilatation) in anaesthetised, vagotomized, or pithed rats (with or without restoration of non-neurogenic vascular tone) by stimulation of endothelial P2Y_1_ receptors [21]. Recent functional evidence from in vivo experimental models underlines that after restoration of vascular tone, this compound subsequently causes a rise in systolic blood pressure (a surrogate for cardiac contractility), which probably depends on the activation of P2Y_1_, P2Y_12_, and P2Y_13_ receptors [21].

Despite these findings, very few publications have addressed the functional capacity of these receptors to modulate neurotransmitter release. For example, Quintas et al. [22] showed that P2Y_1_ and P2Y_12_ receptors prevent sympathetic noradrenaline release in the isolated rat *vas deferens* and tail artery. Moreover, other studies in pithed rats demonstrate distinct receptor mechanisms for ADPβS-induced inhibition, for example: (i) Villanueva-Castillo et al. [23] proved that the cardioaccelerator sympathetic tone is inhibited by P2Y_12_ and probably P2Y_13_ receptors, and (ii) Miguel-Martínez et al. [24] established that inhibition of the vasodepressor sensory CGRPergic tone involves P2Y_1_ and, to a lesser extent, P2Y_13_ receptors via mechanisms independent of ATP-sensitive K^+^ channels.

These prejunctional actions are strongly aligned with parallel in vitro analyses highlighting how intracellular G-protein cascades modulate ionic flux across neural membranes [14]. Nevertheless, to date, no studies in pithed rats have reported whether stimulation of prejunctional P2Y_1_, P2Y_12_, or P2Y_13_ receptors inhibits the vasopressor sympathetic tone or whether this potential sympathoinhibition involves K_ATP_ channels.

Based on these insights, this study pharmacologically examined how ADPβS inhibits the vasopressor sympathetic tone in pithed rats using (i) antagonists at P2Y_1_ (MRS 2500), P2Y_12_ (PSB 0739), and P2Y_13_ (MRS 2211) purinergic receptors (for references, see [1,2,3,5,12]) and (ii) the K_ATP_ channel blocker glibenclamide [24,25,26,27].

We conclude that ADPβS attenuates vasopressor sympathetic activity by activation of sympathoinhibitory P2Y_1_, but not P2Y_12_ or P2Y_13_, receptors, via a K_ATP_ channel-dependent pathway. This approach is relevant for elucidating the neurogenic control of blood pressure regulation, and for identifying potential therapeutic targets in cardiovascular disorders.

## 2. Results

### 2.1. Cardiovascular Variables and Reproducibility of the Vasopressor Responses

Prior to any treatment, the 126 pithed rats had a diastolic blood pressure of 35 ± 1 mmHg and a heart rate of 323 ± 4 beats/min. Moreover, as described earlier [21,23,24], baseline diastolic blood pressure and heart rate did not differ significantly among subgroups (*p* > 0.05). This consistency was observed both during continuous i.v. infusions of vehicle (bidistilled water) or ADPβS, and 10 min after i.v. boluses of vehicle, MRS 2500, PSB 0739, MRS 2211, or glibenclamide.

**Figure 1:**
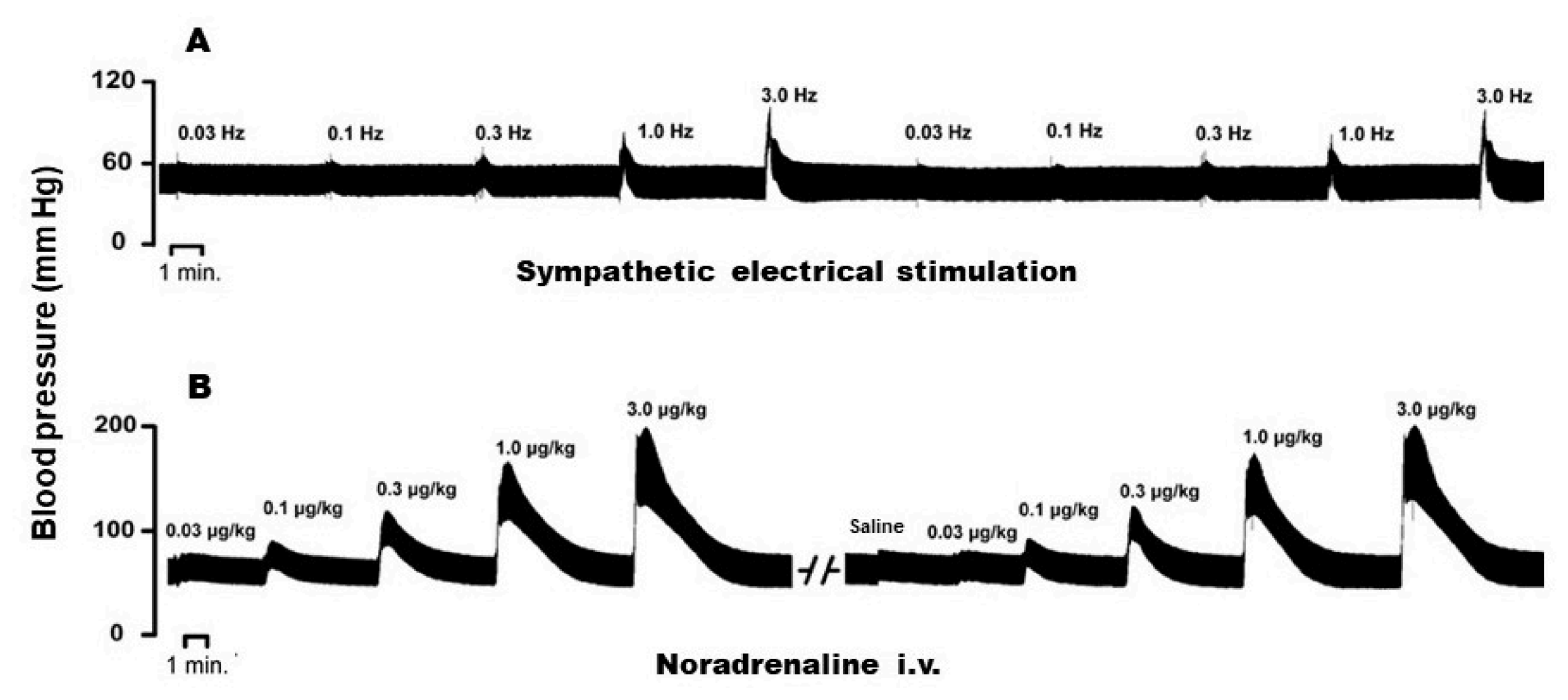
Original recording of the blood pressure responses elicited by electrically stimulating the vasopressor sympathetic tone (**A**) or by administering i.v. boluses of noradrenaline (**B**) under control conditions. (**A**) Electrical stimulation of the vasopressor sympathetic tone at incrementally higher frequencies (0.03–3 Hz) elicits reproducible increases in blood pressure. (**B**) Increasing i.v. doses of noradrenaline (0.03–3 μg/kg) result in reproducible, dose-dependent vasopressor responses (i.e., increases in blood pressure); the dashed line indicates a saline washout period to eliminate the residual noradrenaline from the first D-R curve, whereas the subsequent small response peak (just before 0.03 μg/kg noradrenaline) was elicited by an additional i.v. bolus of 1 mL/kg saline to complete the washout procedure. A stabilization period of 7–10 min was allowed between the completion of the first S-R or D-R curve and the initiation of the second S-R or D-R curve.

Furthermore, Figure 1 shows an original recording of the vasopressor responses elicited by (A) electrically stimulating the sympathetic tone (0.03–3 Hz) and (B) administering noradrenaline i.v. boluses (0.03–3 μg/kg). Both methods generated elevations in blood pressure that were dependent on frequency (A) or dose (B), which proved reproducible during a subsequent stimulus–response (S-R) or dose–response (D-R) curve evaluation.

### 2.2. Effect of Vehicle or ADPβS on the Vasopressor Responses Elicited by Selective Electrical Stimulation

**Figure 2:**
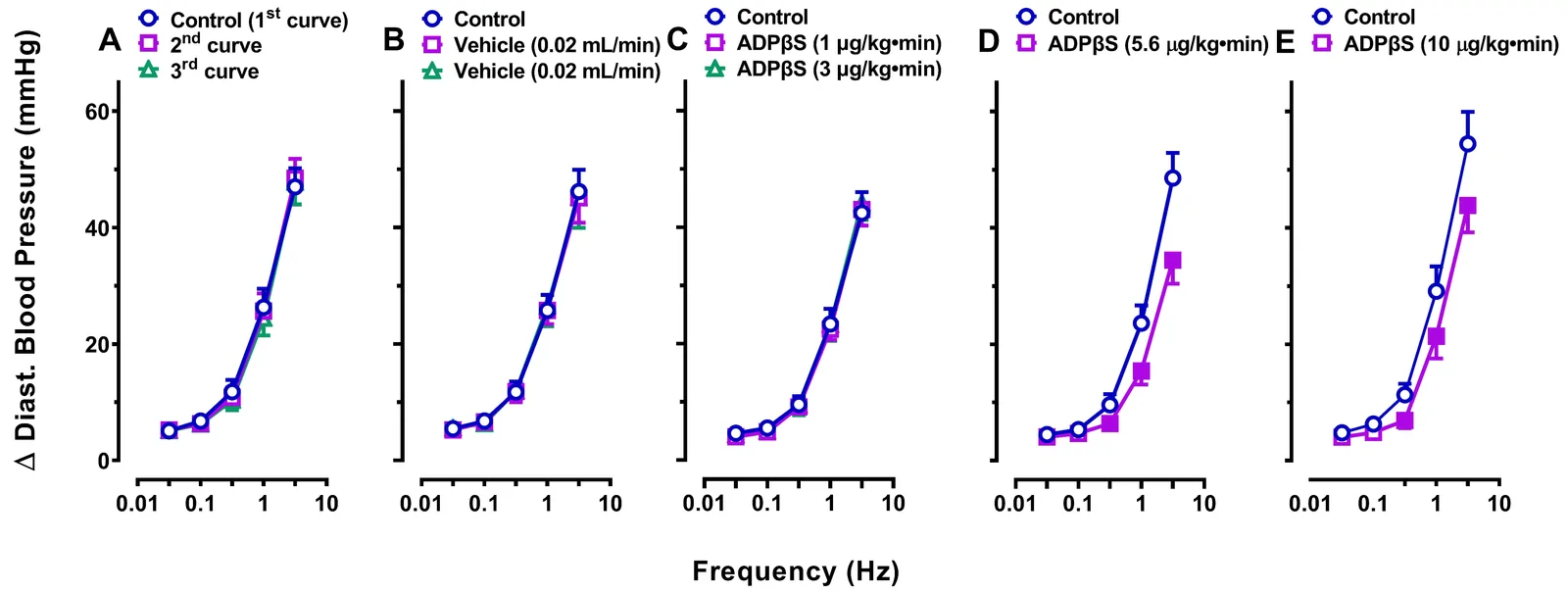
Effect of vehicle or ADPβS infusions on the vasopressor responses elicited by sympathetic stimulation. Effect of (**A**) no treatment, (**B**) vehicle (0.02 mL/min), (**C**) ADPβS (1 and 3 μg/kg·min), (**D**) ADPβS (5.6 μg/kg·min), or (**E**) ADPβS (10 μg/kg·min) on the sympathetically induced elevations in diastolic blood pressure by sympathetic stimulation (*n* = 6 per subgroup). Significant differences from control (*p* < 0.05) are represented by solid squares (■). Δ Diast. Blood Pressure, elevations in diastolic blood pressure.

Figure 2 illustrates the elevations in diastolic blood pressure elicited by stimulation of the vasopressor sympathetic tone before (control) and (A) after two consecutive S-R curves without any treatment with compounds; (B) during two continuous i.v. infusion schemes of the vehicle used to dissolve ADPβS (bidistilled water, 0.02 mL/min); and (C–E) during continuous i.v. infusions of ADPβS at infusion doses of 1 and 3 μg/kg·min (C), 5.6 μg/kg·min (D), and 10 μg/kg·min (E).

As established in previous studies [28,29,30,31,32,33], the sympathetically induced vasopressor responses were not altered (*p* > 0.05) after two consecutive S-R curves in untreated animals (Figure 2A), or in animals infused with bidistilled water (Figure 2B); this finding confirms the high reproducibility of the vasopressor responses. Furthermore, the sympathetic vasopressor responses (i) were not significantly changed (*p* > 0.05) during the continuous i.v. infusions of ADPβS at 1 and 3 μg/kg·min (Figure 2C) and (ii) were significantly attenuated by ADPβS at the infusion doses of 5.6 and 10 μg/kg·min (*p* < 0.05), although the inhibition induced by 5.6 μg/kg·min ADPβS (Figure 2D) was not significantly different (*p* > 0.05) from that induced by 10 μg/kg·min ADPβS (Figure 2E). Clearly, this ADPβS-induced inhibition did not depend on the dose used, as observed in other studies using the same infusion doses [24]. Based on this finding, we selected an infusion dose of 5.6 μg/kg·min ADPβS for further analysis with P2Y purinergic receptor antagonists to identify the specific receptor profile involved.

### 2.3. Effect of the Vehicle or of the Antagonists Per Se at P2Y_1_ (MRS 2500), P2Y_12_ (PSB 0739), or P2Y_13_ (MRS 2211) Purinergic Receptors on Sympathetic Vasopressor Responses During a Continuous Infusion of the Vehicle

To guide our in vivo pharmacological characterisation, the selection of the selective P2Y receptor ligands and their respective doses was based on their known interaction kinetics. Accordingly, Table 1 outlines the potency and affinity data (reported as *p*IC_50_, *p*EC_50_, and *p*K_i_ values) of these compounds for human P2Y_1_, P2Y_12_, and P2Y_13_ receptors, adapted from a previous study [24]. While these parameters reflect binding affinity rather than functional efficacy in vivo, they provide the quantitative basis necessary to ensure complete receptor blockade in our experimental setup.

**Figure 3:**
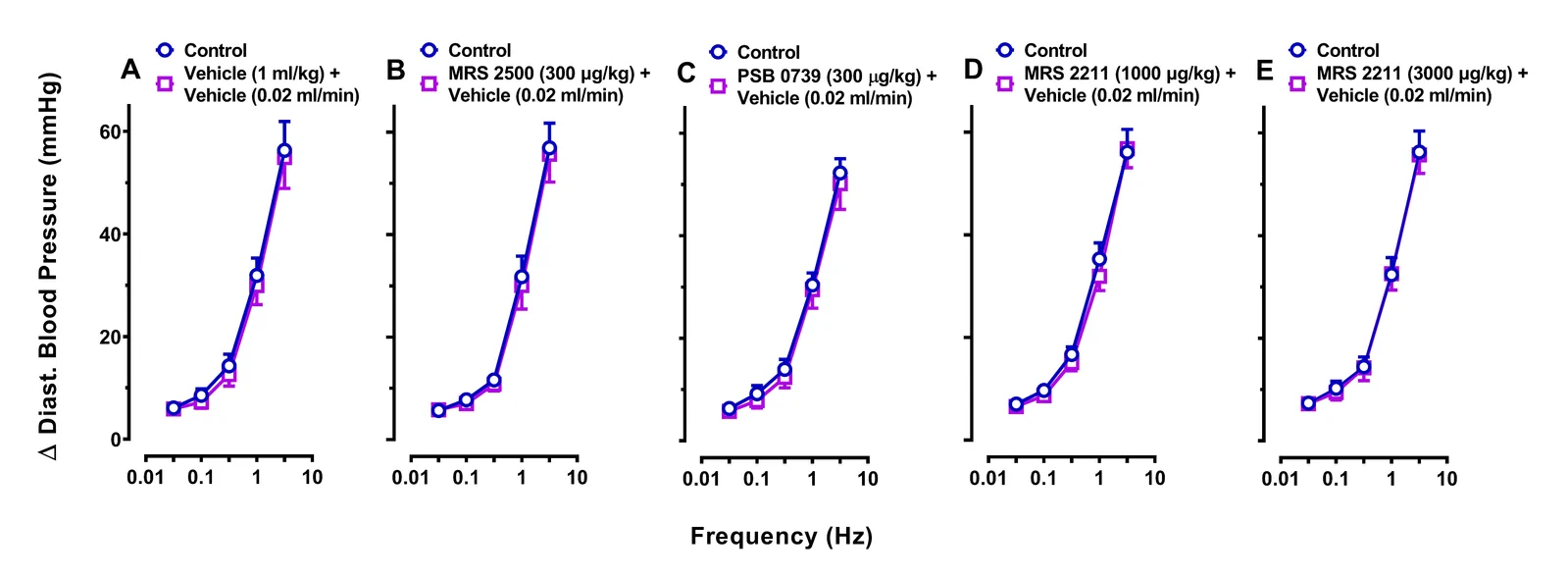
Effect of the vehicle or of the antagonists themselves at P2Y purinergic receptors on the vasopressor responses evoked by sympathetic stimulation. Sympathetically evoked elevations in diastolic blood pressure before (control) and after i.v. administration of (**A**) vehicle (1 mL/kg); (**B**) MRS 2500 (300 μg/kg); (**C**) PSB 0739 (300 μg/kg); (**D**) MRS 2211 (1000 μg/kg); and (**E**) MRS 2211 (3000 μg/kg) (*n* = 6 in each subgroup). Δ Diast. Blood Pressure, elevations in diastolic blood pressure.

To achieve accurate pharmacological evaluation of the receptors that participate in ADPβS-induced inhibition of sympathetic vasopressor responses, a critical control is that administering a vehicle or a P2Y antagonist alone did not affect the sympathetically induced vasopressor responses per se. Figure 3 depicts the vasopressor responses elicited by sympathetic stimulation before (control group) and after i.v. administration of the vehicle (bidistilled water; 1 mL/kg) or the antagonists at P2Y_1_ (MRS 2500), P2Y_12_ (PSB 0739), or P2Y_13_ (MRS 2211) receptors during an infusion with bidistilled water (0.02 mL/min).

These responses were not significantly altered (*p* > 0.05) after vehicle (1 mL/kg, Figure 3A), MRS 2500 (300 µg/kg, Figure 3B), PSB 0739 (300 µg/kg, Figure 3C), or MRS 2211 (1000 and 3000 µg/kg, Figure 3D and Figure 3E, respectively) given i.v. Similarly, baseline diastolic blood pressure and heart rate values remained without significant changes (*p* > 0.05) after administration of these compounds. These results suggest that the doses of the administered compounds failed to exert direct effects on sympathetic vasopressor responses themselves. For further considerations, see Section 3.3.

### 2.4. Effect of the Antagonists at P2Y_1_ (MRS 2500), P2Y_12_ (PSB 0739), or P2Y_13_ (MRS 2211) Purinergic Receptors on the Vascular Sympathoinhibition Induced by ADPβS

**Figure 4:**
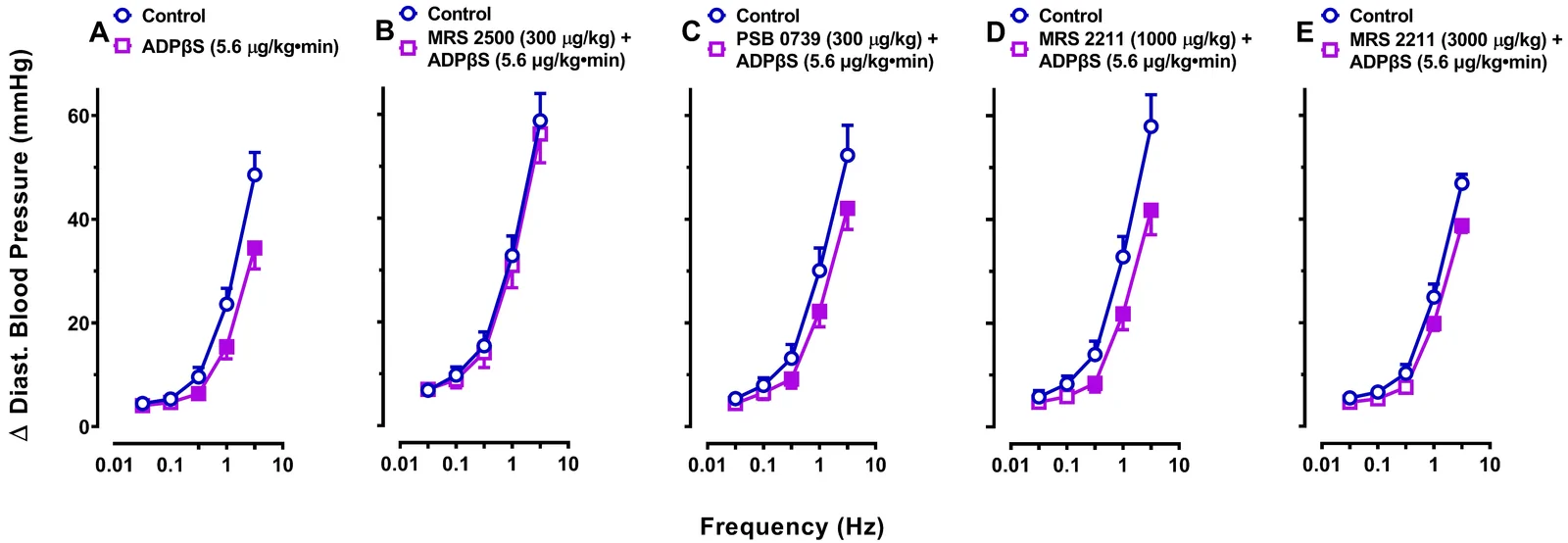
Effect of P2Y receptor antagonists on ADPβS-induced vascular sympathoinhibition. Effect of (**A**) vehicle (1 mL/kg); (**B**) MRS 2500 (300 μg/kg); (**C**) PSB 0739 (300 μg/kg); (**D**) MRS 2211 (1000 μg/kg); and (**E**) MRS 2211 (3000 μg/kg) given i.v. (*n* = 6 per subgroup) on the vascular sympathoinhibition by ADPβS (5.6 μg/kg·min). Solid squares (■) indicate *p* < 0.05 vs. control. Note that (**A**) replicates the results shown in Figure 2D to minimize animal use, in view that (**C**,**D**) display similar results. Δ Diast. Blood Pressure, elevations in diastolic blood pressure.

After confirming that neither the vehicle nor the P2Y receptor antagonists alone affected sympathetic vasopressor responses (Figure 3), we evaluated these responses before (control group) and after i.v. administration of the antagonists for P2Y_1_ (MRS 2500), P2Y_12_ (PSB 0739), and P2Y_13_ (MRS 2211) purinergic receptors followed by a continuous i.v. infusion of 5.6 µg/kg·min ADPβS (Figure 4). The ADPβS-induced vascular sympathetic inhibition in untreated animals (Figure 4A): (i) remained unchanged after PSB 0739 (300 µg/kg, Figure 4C), or MRS 2211 (1000 and 3000 µg/kg, Figure 4D and Figure 4E, respectively) given i.v.; (ii) was abolished after 300 µg/kg MRS 2500 given i.v. (Figure 4B), reinforcing the involvement of P2Y_1_ receptors.

### 2.5. Effect of the Vehicle or of the K_ATP_ Channel Blocker Glibenclamide on Sympathetic Vasopressor Responses and/or on the Sympathoinhibition Elicited by ADPβS

**Figure 5:**
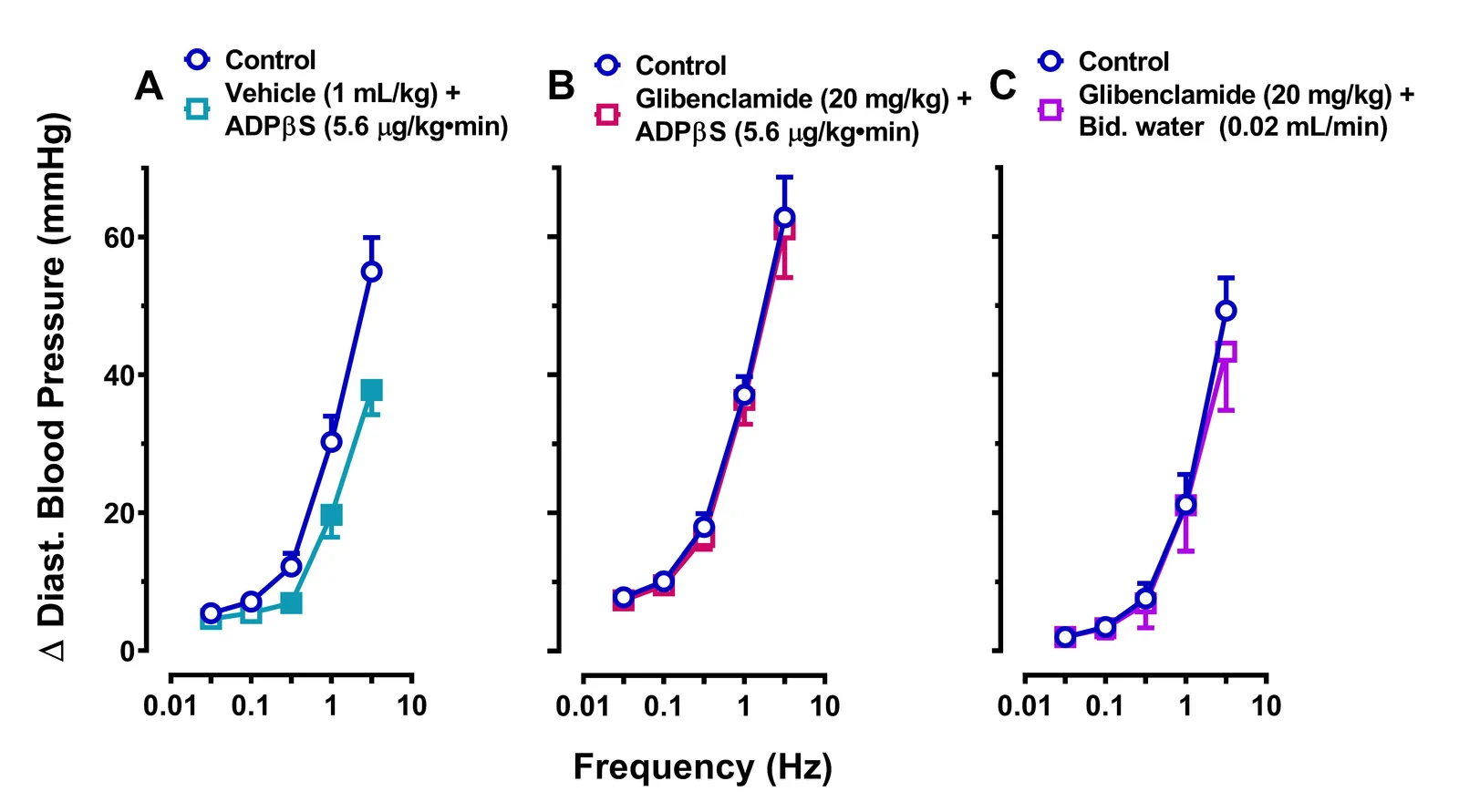
Effect of the vehicle or of the K_ATP_ channel blocker glibenclamide on ADPβS-induced vascular sympathoinhibition. Sympathetic vasopressor responses before and after an i.v. bolus of (**A**) the vehicle used to dissolve glibenclamide (1 mL/kg) followed by ADPβS (5.6 μg/kg·min, i.v.); (**B**) glibenclamide (20 mg/kg) followed by ADPβS (5.6 μg/kg·min, i.v.); and (**C**) glibenclamide (20 mg/kg) followed by the vehicle for ADPβS (0.02 mL/min, i.v.) (*n* = 6 per subgroup). Significant differences from control (*p* < 0.05) are represented by solid squares (■). Δ Diast. Blood Pressure, elevations in diastolic blood pressure; Bid. water, bidistilled water.

This experimental protocol investigated the possible participation of K_ATP_ channels in the vascular sympathoinhibition elicited by ADPβS. We first ensured that the administration of the vehicle or blocker alone did not affect the sympathetic vasopressor responses themselves, as detailed in Section 2.3. Figure 5 illustrates these vasopressor responses before and after an i.v. bolus of (A) the vehicle used to dissolve glibenclamide (a mixture of 34% NaOH 0.2 M, 33% polyethylene glycol [PEG], and 33% ethanol; 1 mL/kg) followed by ADPβS (5.6 μg/kg·min, i.v.); (B) glibenclamide (20 mg/kg) followed by ADPβS (5.6 μg/kg·min, i.v.); and (C) glibenclamide (20 mg/kg) followed by the vehicle for ADPβS (0.02 mL/min, i.v.).

As shown in Figure 5, the ADPβS-induced sympathoinhibition (i) remained unchanged by the vehicle for glibenclamide (1 mL/kg; Figure 5A), as observed with other treatments in this study (see Figure 2A and Figure 4C,D), and (ii) was abolished by glibenclamide (20 mg/kg; Figure 5B) in its function as a K_ATP_ channel blocker. These findings suggest that the ADPβS-induced inhibition of sympathetic vasopressor responses involves K_ATP_ channel-dependent mechanisms. Conversely, the fact that this dose of glibenclamide had no effect on sympathetic vasopressor responses themselves (Figure 5C) indicates that the effect shown in Figure 5B was not masked or confounded by a direct effect of glibenclamide on these responses (for further details, see Section 3).

### 2.6. Comparison of the Profile of Blockade Elicited by the P2Y Receptor Antagonists, Glibenclamide, or the Vehicles on the Sympathoinhibition Evoked by ADPβS

We compared the profile of blockade elicited by the antagonists or glibenclamide on ADPβS-induced sympathoinhibition (Figure 4B–E) with that produced by ADPβS alone in untreated animals (Figure 4A). The results depicted in Figure 4A replicate those shown in Figure 2D to minimise the use of animals, in view of the fact that Figure 4C,D display similar results. In other words, the sympathoinhibition induced by ADPβS after administration of the antagonists MRS 2500 or PSB 0739 was not significantly different (*p* > 0.05) from that induced by ADPβS in untreated animals, assuming that ADPβS would have produced the same results after vehicle (1 mL/kg, i.v.). Clearly, this was the case with the results shown in Figure 5A with the vehicle used to dissolve glibenclamide. Indeed, the sympathoinhibition by ADPβS after administration of the vehicle for glibenclamide was not significantly different (*p* > 0.05) from that produced by ADPβS in untreated animals. Accordingly, the evaluation of the effects of 1 mL/kg of bidistilled water on the sympathoinhibition by ADPβS was omitted. On the other hand, when comparing Figure 4E with Figure 4A and Figure 5A, it is clear that 3000 μg/kg MRS 2211 blocked the sympathoinhibition induced by ADPβS at 0.3 Hz (*p* > 0.05).

### 2.7. Effect of ADPβS on the Vasopressor Responses Elicited by Exogenous Noradrenaline

**Figure 6:**
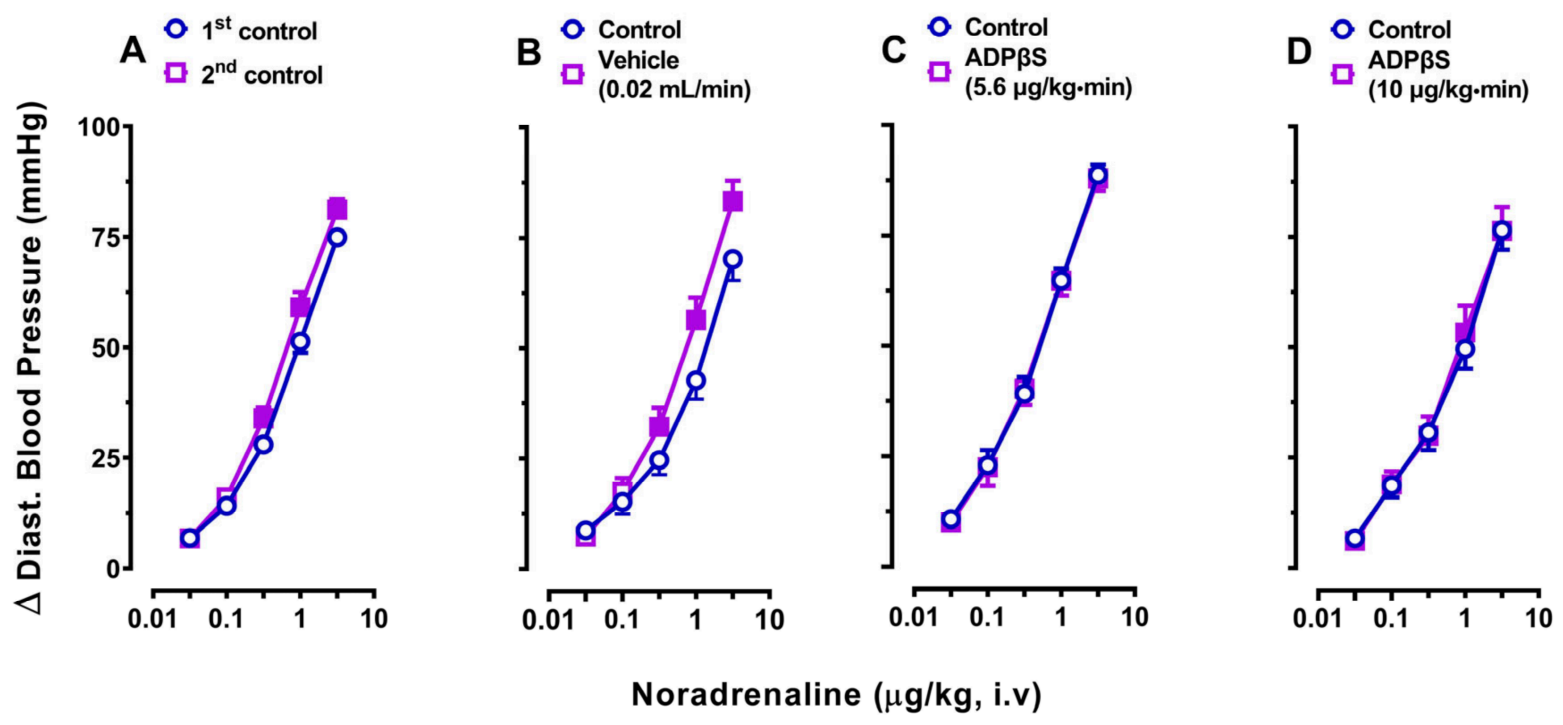
Effect of ADPβS on vasopressor responses to noradrenaline. Vasopressor responses produced by i.v. boluses of noradrenaline before and after i.v. administration of (**A**) nothing (2nd control); or (**B**) vehicle (0.02 mL/min, i.v.), (**C**) ADPβS (5.6 μg/kg·min, i.v.), and (**D**) ADPβS (10 μg/kg·min, i.v.) (*n* = 6 in each subgroup). Significant differences from control (*p* < 0.05) are represented by solid squares (■). Δ Diast. Blood Pressure, elevations in diastolic blood pressure.

Although the results in Figure 2D,E clearly demonstrate that ADPβS inhibited the vasopressor sympathetic tone, it was necessary to exclude potential systemic interactions of this long-lasting agonist at the vascular level, such as with the vasopressor responses to exogenous noradrenaline. Hence, Figure 6 displays these responses in animals: (A) without treatment; or receiving (B) vehicle (0.02 mL/min, i.v.), (C) ADPβS (5.6 μg/kg·min, i.v.), or (D)ADPβS (10 μg/kg·min, i.v.). These responses were not attenuated, but rather weakly potentiated (*p* < 0.05) in the second D-R curve to noradrenaline in untreated animals (Figure 6A) and in animals infused with bidistilled water (Figure 6B). This slight potentiation could be attributed to the potential saturation of neuronal and extraneuronal noradrenaline reuptake mechanisms. Importantly, noradrenaline-induced vasopressor responses remained practically unaffected (*p* > 0.05) during ADPβS infusions at 5.6 μg/kg·min (Figure 6C) and 10 μg/kg·min (Figure 6D).

## 3. Discussion

### 3.1. General

As previously established [5,34,35,36], vascular tone (i) regulates blood pressure by determining peripheral vascular resistance and (ii) is modulated by a diverse array of receptors located across blood vessels, perivascular nerves, autonomic ganglia, and the CNS. Within this context, prejunctional purinergic receptors on perivascular sympathetic nerves modulate noradrenaline release, with P2X receptors facilitating [37] and P2Y receptors inhibiting this process [3,5]. However, limited research has investigated the participation of sympathoinhibitory P2Y receptors in the systemic resistance vasculature, i.e., the primary determinants of blood pressure (for references, see [1]).

As recently reviewed [5], pithed rats lack central baroreflex mechanisms but retain intact peripheral neurogenic (i.e., autonomic and sensory) functions, making this model a fundamental tool for investigating the peripheral cardiovascular responses to ADPβS. Therefore, this study evaluated the pharmacological properties of the P2Y purinergic receptors that inhibit the rat vasopressor sympathetic tone by quantifying the increases in diastolic blood pressure (a marker of systemic vascular tone [38]) driven by selective sympathetic vasopressor stimulation [28,29,30,31,32,33]. For this purpose, we used ADPβS, as it selectively stimulates P2Y_1_, P2Y_12_ and P2Y_13_ purinergic receptors (for references, see [1,2,3,5,12]).

Our results suggest that ADPβS inhibits the vasopressor sympathetic tone (Figure 4A) by activating P2Y_1_ receptors (Figure 4B). This sympathoinhibitory action of ADPβS (i) involves K_ATP_ channel-dependent mechanisms (Figure 5B), without pharmacological evidence for the participation of P2Y_12_ or P2Y_13_ receptors (Figure 4C–E), and (ii) is mediated at the prejunctional level, given that the vasopressor responses to exogenous noradrenaline were not attenuated (Figure 6C,D). Interestingly, the pharmacological features of these P2Y_1_ receptors (i) are similar to those of the prejunctional P2Y (mainly P2Y_1_) receptors that inhibit the vasodepressor sensory CGRPergic tone [24] or the endothelial P2Y (mainly P2Y_1_) receptors that mediate systemic vasodilatation by decreasing diastolic blood pressure [21], and (ii) contrast with those of the prejunctional P2Y (primarily P2Y_12_ and, less prominently, P2Y_13_) receptors that elicit cardiac sympathetic inhibition [23].

Since all findings were obtained in healthy male Wistar pithed rats, the pharmacological profiles of the prejunctional P2Y receptors across both sympathetic pathways would be expected to be similar. However, this discrepancy in the prejunctional receptors inhibiting the vasopressor (P2Y_1_) and cardioaccelerator (P2Y_12/13_) sympathetic pathways has previously been attributed to differences in the spinal anatomical origin of the preganglionic neurons [33,39]. Indeed, in pithed rats, the vasopressor sympathetic tone arises from the spinal T_7_–T_9_ segments [33], whereas the cardioaccelerator sympathetic tone originates from the spinal C_7_–T_1_ segments [39].

In contrast, the vasodepressor sensory CGRPergic tone arises from the spinal T_9_–T_12_ segments [40] and, like the vasopressor sympathetic tone (T_7_–T_9_; as described above), is also inhibited by prejunctional P2Y_1_ receptors [24]. However, unlike ADPβS-induced inhibition of the vasopressor sympathetic tone, which is reversed by glibenclamide (involving K_ATP_ channel-dependent mechanisms; Figure 5B), the ADPβS-induced inhibition of the vasodepressor sensory CGRPergic tone is resistant to glibenclamide [24].

This comparison highlights a sophisticated level of functional heterogeneity in peripheral purinergic transduction mechanisms. It demonstrates that even within the same animal model, identical prejunctional receptor subtypes (i.e., P2Y_1_) can recruit different intracellular cascades depending on the specific physiological nature and anatomical origin of the nerve terminals.

### 3.2. Systemic Vascular Sympathoinhibition Elicited by ADPβS

ADPβS has been widely employed as a pharmacological tool to identify P2Y purinergic receptors (for references, see [5]). As a stable analogue of ADP, it exhibits affinity for P2Y_1_, P2Y_12_, and P2Y_13_ receptors, as summarized in Table 1 (located in Section 2.3). Therefore, its use in this study ensured the activation of these receptors to verify their presence and functional role in modulating the vasopressor sympathetic tone.

Our results show that only the highest infusion doses of ADPβS (i.e., 5.6 and 10 μg/kg·min) were capable of inhibiting the vasopressor sympathetic tone (Figure 2D,E), consistent with findings previously reported for the vasodepressor sensory CGRPergic tone [24]. The lack of a significant difference (*p* > 0.05) in the sympathoinhibition between the 5.6 and 10 μg/kg·min doses of ADPβS suggests potential receptor desensitization, a phenomenon previously reported for endothelial P2Y_1_ receptors [20]. In support of this hypothesis, we also observed that 5.6 and 10 μg/kg·min ADPβS elicited a short-lasting, mild hypotension that resolved within 3–5 min. This transient systemic effect, also noted in a previous study [24], occurred independently of the inhibition of sympathetic vasopressor responses (Figure 2D,E) and was not evaluated further.

Based on the affinity profile of ADPβS for P2Y_1_, P2Y_12_, and P2Y_13_ purinergic receptors (Table 1), the above findings suggest that its effective infusion doses (5.6 and 10 μg/kg·min) generated sufficient concentrations at the neurovascular junction to activate these receptors. Further pharmacological evaluation with selective P2Y receptor antagonists (Table 1) was required to identify the precise subtype(s) mediating this response (see below).

### 3.3. Possible Participation of P2Y_1_, P2Y_12_, or P2Y_13_ Receptors in the Systemic Vascular Sympathoinhibition Elicited by ADPβS

Previous studies in pithed rats have demonstrated that the inhibition of the vasopressor sympathetic tone can be mediated by the stimulation of specific prejunctional G_i/o_-coupled receptors, such as serotonin 5-HT_1A/1B/1D_ [31], dopamine D_3/4_ [33], cannabinoid CB_1_ [41], and adrenergic α_2A/2B/2C_ [42]. After verifying that the vehicle, MRS 2500, PSB 0739, and MRS 2211 did not alter control sympathetic vasopressor responses (Figure 3), we demonstrated that the ADPβS-induced inhibition of these responses was completely blocked by MRS 2500 but remained essentially unaffected by PSB 0739 or MRS 2211 (Figure 4). These findings strongly support the involvement of P2Y_1_ receptors in this sympathoinhibitory mechanism. This interpretation is further supported by the crucial facts that these antagonists (i) were administered at doses known to fully block their respective P2Y_1_, P2Y_12_, and P2Y_13_ cardiovascular targets in pithed rats [21,23,24] and (ii) exhibit differential affinity profiles consistent with their receptor selectivity (Table 1).

From a receptor-kinetics perspective, the observation that these antagonists failed to alter baseline haemodynamic values or sympathetic vasopressor responses per se (Figure 3) is entirely consistent with the classical receptor theory. Accordingly, since a competitive antagonist lacks intrinsic activity (i.e., efficacy = 0), its blockade remains “silent” in the absence of an active ligand [43]. In our pithed rat model, this lack of independent effects probably reflects (i) the absence of a tonically active endogenous purinergic tone at these prejunctional neurovascular terminals and/or (ii) a lack of constitutive (spontaneous) activity of prejunctional P2Y_1_, P2Y_12_, and P2Y_13_ receptors under baseline conditions.

Conversely, the pharmacological profile shifts completely during continuous ADPβS infusion (Figure 4). Once ADPβS binds to prejunctional P2Y_1_ receptors to inhibit noradrenaline release, MRS 2500 exerts its blocking capacity by directly competing for the orthosteric binding site, successfully abolishing the sympathoinhibition (Figure 4B). Meanwhile, because P2Y_12_ and P2Y_13_ receptors are not involved in this cascade, their respective silent antagonists (i.e., PSB 0739 and MRS 2211) remain without effect (Figure 4C–E). These receptor-mediated actions line up with the complex intracellular G-protein signalling cascades and cellular transduction pathways outlined in the Introduction, which are known to orchestrate overall vascular neuro-effector transmission [17,18,19].

### 3.4. Role of K_ATP_ Channels in the Inhibition of Vasopressor Sympathetic Tone by ADPβS

K_ATP_ channels modulate vascular tone by inducing hyperpolarisation of vascular smooth muscle cells, thereby linking them to P2Y_1_ receptor signalling. For this reason, we analysed the effects of glibenclamide to ascertain the specific role of K_ATP_ channels in the inhibition by ADPβS of the vasopressor sympathetic responses [36,44,45]. It is important to note that, under our experimental conditions, glibenclamide alone failed to significantly alter baseline haemodynamic values or vasopressor sympathetic responses (Figure 5C). In keeping with these findings, other studies have reported that, under basal resting conditions, K_ATP_ channels in both perivascular sympathetic nerve terminals and vascular smooth muscle are functionally closed or silent [46]. Consequently, administering a channel blocker when the channels are already inactive exerts no direct effect on baseline sympathetic tone, which explains why glibenclamide per se failed to modify these vasopressor responses (Figure 5C).

These findings align with the established mechanisms of P2Y_1_ purinergic receptors [47,48,49]. The ADPβS activation of prejunctional P2Y_1_ purinergic receptors may elicit vascular sympathoinhibition via a mechanism that involves activation of K_ATP_ channels, leading to hyperpolarisation of sympathetic nerve terminals (Figure 5B). Concurrently, endothelial P2Y_1_ receptors activate phospholipase C and promote nitric oxide formation, thereby increasing guanylate cyclase activity; this cascade may also involve the opening of K_ATP_ channels to induce vasorelaxation [47,48,49].

This purinergic pathway modulates perivascular sensory neurotransmission independently of K_ATP_ channels [24]. In parallel, other lines of evidence confirm that glibenclamide reverses the prejunctional inhibition of noradrenaline release in distinct peripheral sympathetic nerve endings, where endogenous purinergic co-transmission controls cardiovascular neurotransmitter output [50,51]. These studies strongly support our novel findings (Figure 5B) by validating that prejunctional purinergic signals and downstream K_ATP_ channels operate as a fundamental feedback mechanism to fine-tune sympathetic transmission at the systemic neurovascular level.

To integrate these mechanisms within a broader systemic context, the recruitment of K_ATP_ channels may act as a pivotal link coupling local purinergic receptor activation with global haemodynamic control. At the neuronal level, these channels are known to modulate noradrenergic transmission, as demonstrated by pioneering in vivo evidence in rats where glibenclamide blocks the K_ATP_ channel-mediated attenuation of sympathetic vasoconstrictor responses [52]. By reducing this noradrenergic neurovascular drive across the peripheral resistance vasculature, where potassium currents orchestrate overall cell hyperpolarisation and blood vessel diameter [53], this purinergic P2Y_1_–K_ATP_ prejunctional cascade could operate as an essential homeostatic pathway to restrict excessive vasoconstriction and modulate overall arterial blood pressure. Contextualising our novel findings within this unified framework underscores how discrete prejunctional molecular interactions may directly shape systemic vascular resistance under integrative physiological conditions.

### 3.5. Blockade Profiles of P2Y Receptor Antagonists or Glibenclamide vs. Vehicle

A comparison of the blockade profiles produced by the P2Y receptor antagonists or glibenclamide vs. their corresponding vehicles on the sympathoinhibition by ADPβS (specifically comparing Figure 4E with Figure 4A and Figure 5A) revealed that 3000 μg/kg MRS 2211 only reversed this sympathoinhibition at 0.3 Hz (*p* > 0.05; Figure 4E). However, this finding does not provide strong pharmacological support for P2Y_13_ receptor involvement when considering the binding affinities of MRS 2211 for P2Y receptor subtypes (Table 1) and the doses used.

The limited blockade by MRS 2211 at 3000 μg/kg (Figure 4E), together with its lack of effect at 1000 μg/kg (Figure 4D), might reflect its moderate affinity for P2Y_1_ receptors (Table 1). As pointed out by Miguel-Martínez et al. [24], “if P2Y_13_ receptors played a significant role, 1000 and 3000 μg/kg MRS 2211 would be expected to produce a clear reversal of the ADPβS-induced inhibition, similar to that observed with MRS 2500” (Figure 4B). Most importantly, in another study in anaesthetised rats, where P2Y_13_ receptors play a role, 1000 μg/kg MRS 2211 reversed the ADPβS-induced inhibition of CGRP release from meningeal sensory neurons [54].

In summary, the systemic vascular sympathoinhibition elicited by ADPβS clearly involves prejunctional P2Y_1_ receptors. The apparently negligible role of P2Y_13_ receptors (if any), based on the blockade profile (Figure 4E) and binding properties (Table 1) of MRS 2211, seems rather limited, and its future validation will depend on the availability of a highly selective P2Y_13_ receptor antagonist.

### 3.6. Prejunctional Nature of the Inhibition by ADPβS of Sympathetic Vasopressor Responses

As described in Section 2.7, when comparing the first and second D-R curves to exogenous noradrenaline, a slight but significant potentiation (*p* < 0.05) of the vasopressor responses was induced when producing a second D-R curve under control conditions (Figure 6A,B). Interestingly, this potentiation was practically abolished in animals infused with 5.6 or 10 μg/kg·min ADPβS (Figure 6C and Figure 6D, respectively).

Similar phenomena have been observed by other investigators utilizing this experimental model [55]. A plausible hypothesis is that this apparent potentiation may involve sensitization of α_1_-adrenoceptors and/or local accumulation of noradrenaline at the sympathetic neurovascular junction due to the transient saturation of neuronal and extraneuronal reuptake mechanisms. In turn, during subsequent administrations, this saturation could lead to higher local concentrations of noradrenaline at adrenergic receptors, thereby enhancing the vasoconstrictor response.

Given that this reuptake mechanism appears to be saturated during the second D-R curve to noradrenaline (Figure 6C,D), the infusions of ADPβS could have counteracted this slight potentiation relative to the first D-R curve via the activation of endothelial P2Y_1_ receptors. This activation results in a systemic vasodilator (vasodepressor) response [21] that may have overshadowed the enhanced vasoconstriction elicited by exogenous noradrenaline. Supplementary experiments revealed that the enhanced vasopressor responses to noradrenaline were restored to control values when eliciting a third and fourth D-R curve during ADPβS infusions; this recovery is potentially attributable to the desensitization of endothelial P2Y_1_ receptors [20].

In short, even after considering the weak systemic vasodilatation elicited during the ADPβS infusions (mediated by endothelial P2Y_1_ receptors in pithed rats [21]), these findings reinforce the view that the inhibition by ADPβS of the sympathetic vasopressor responses is predominantly prejunctional in nature. This is supported by the observation that these infusion doses elicited inhibition of the sympathetic vasopressor responses (Figure 2D,E) without attenuating those to exogenous noradrenaline (Figure 6C,D).

### 3.7. Limitations of the Study and Methodological Considerations

Admittedly, this study has several limitations and methodological considerations.

First, the systemic nature of i.v. administration in pithed rats may introduce pharmacokinetic confounders, such as rapid metabolism or clearance, as previously considered for this experimental model [23,33]. Nevertheless, continuous i.v. infusions (rather than i.v. boluses or non-vascular routes) remain the gold standard in this setup. This continuous approach is strictly necessary to establish and maintain a constant plateau of prejunctional inhibition, which is essential for accurately constructing full S-R or D-R curves [24,28,29,30,31,32,33]. Alternative non-vascular routes, such as intraperitoneal or oral administration, would introduce absorption-related confounding variables that drastically alter the onset, duration, and magnitude of the drug’s effect, making the quantification of these steady-state receptor-mediated changes unfeasible [56]. Consequently, these potential confounders inherent to i.v. administration do not invalidate our core results, instead providing critical insights into the functional sympathoinhibitory role of P2Y_1_ receptors located on systemic vascular (vasopressor) sympathetic nerves.

Second, the binding affinities displayed in Table 1 were obtained from human rather than rodent P2Y receptors. Nonetheless, earlier findings demonstrate that it is highly feasible to extrapolate these binding profiles across species [24].

Finally, a definitive explanation for the precise mechanisms underlying the transient potentiation of noradrenaline responses, and its subsequent attenuation during ADPβS infusions, remains to be fully elucidated. While our findings point towards the saturation of noradrenaline reuptake mechanisms and the subsequent desensitization of endothelial P2Y_1_ receptors, additional experiments outside the scope of the present study are warranted to definitively validate these hypotheses.

### 3.8. Perspectives and Potential Clinical Significance

Several studies highlight the potential of P2Y receptors as therapeutic targets for cardiovascular pathologies, including hypertension, ischemic injury, coronary vasospasm, and thromboembolic diseases (for references, see [2,5,18,19]). Our pharmacological evidence showing that the ADPβS-induced inhibition of the vasopressor sympathetic tone involves prejunctional P2Y_1_ receptors, via K_ATP_ channel-dependent mechanisms, helps elucidate the neurogenic control of vascular resistance and blood pressure regulation. Accordingly, the development of selective P2Y_1_ receptor agonists may offer potential therapeutic utility for the management of resistant arterial hypertension, where sympathetic overactivity is the core pathophysiological substrate [57]. Conversely, when developing P2Y_1_ receptor antagonists, the blockade of prejunctional and endothelial P2Y_1_ receptors must be evaluated as a potential source of cardiovascular side effects.

From another perspective, two critical avenues emerge for future research, namely (i) a comparative assessment of the relative contributions of prejunctional P2Y_1_ receptors (which mediate systemic vascular sympathoinhibition) and endothelial P2Y_1_ receptors (which mediate vasodilatation) and (ii) the exploration of P2Y_1_ receptor expression and activity in perivascular sympathetic neurons under pathological, rather than exclusively physiological, conditions.

## 4. Materials and Methods

### 4.1. Experimental Animals

As reported in previous studies [21,23,24,28,29,30,31,32,33,58], animal experiments were approved by our Institutional Ethics Committee CICUAL-Cinvestav (protocol 0139-15) and complied with the Mexican Official Norm NOM-062-ZOO-1999 [59], the US Guide [60], and the ARRIVE guidelines [61].

### 4.2. General Methods

The experimental sample consisted of 126 healthy male Wistar rats (body weight: 250–280 g) provided by our institutional animalarium. As described earlier [21,23,24,28,29,30,31,32,33,58], “all animals were maintained in a room at 50% humidity, a 12/12 h light/dark cycle (light beginning at 07:00 h) and a temperature of 22 ± 2 °C, with food and water ad libitum. The animals were anaesthetised intraperitoneally with sodium pentobarbital (60 mg/kg)”.

Once the animals were anaesthetised, and after confirming the absence of corneal and pedal reflexes, as well as the tail flexion response to pressure, a tracheostomy was performed. This consisted of inserting a polyethylene cannula into the trachea, which was connected to a Ugo Basile model 7025 ventilation pump to provide artificial ventilation at a rate of 56 cycles/min and a stroke volume of 20 mL/kg [62].

As previously established [28,29,30,31,32,33], “the body temperature of the animals was maintained at 37 °C by using a lamp and monitored with a rectal thermometer. At this point, the rats were pithed by inserting a stainless steel rod through the ocular orbit and foramen magnum into the vertebral foramen [63]”. This was performed for two main reasons, namely (i) to destroy the CNS and thus prevent any regulatory baroreflex activity and (ii) to allow the replacement of the rod with an electrode that selectively stimulates the T_7_–T_9_ spinal segments to drive the sympathetic vasopressor tone [28,29,30,31,32,33].

As noted before [28,29,30,31,32,33], “a bilateral cervical vagotomy was subsequently performed, and polyethylene catheters were inserted into: (i) the left femoral vein for the administration of a continuous i.v. infusion of vehicle (bidistilled water) or ADPβS; and (ii) the right femoral vein for the administration of i.v. boluses of vehicle, purinergic antagonists, or exogenous noradrenaline. For the doses of compounds and other details, see Section 4.3 (Experimental Protocols) described below. In addition, the left carotid artery was cannulated with a polyethylene catheter connected to a TSD104A blood pressure transducer (Biopac Systems Inc., Goleta, CA, USA) to record blood pressure and heart rate. Both parameters were recorded simultaneously using a data acquisition unit (MP150A-CE, Biopac Systems Inc., Goleta, CA, USA) and processed with Acqknowledge v4.2.0 software (Biopac Systems Inc., Goleta, CA, USA)”.

**Figure 7:**
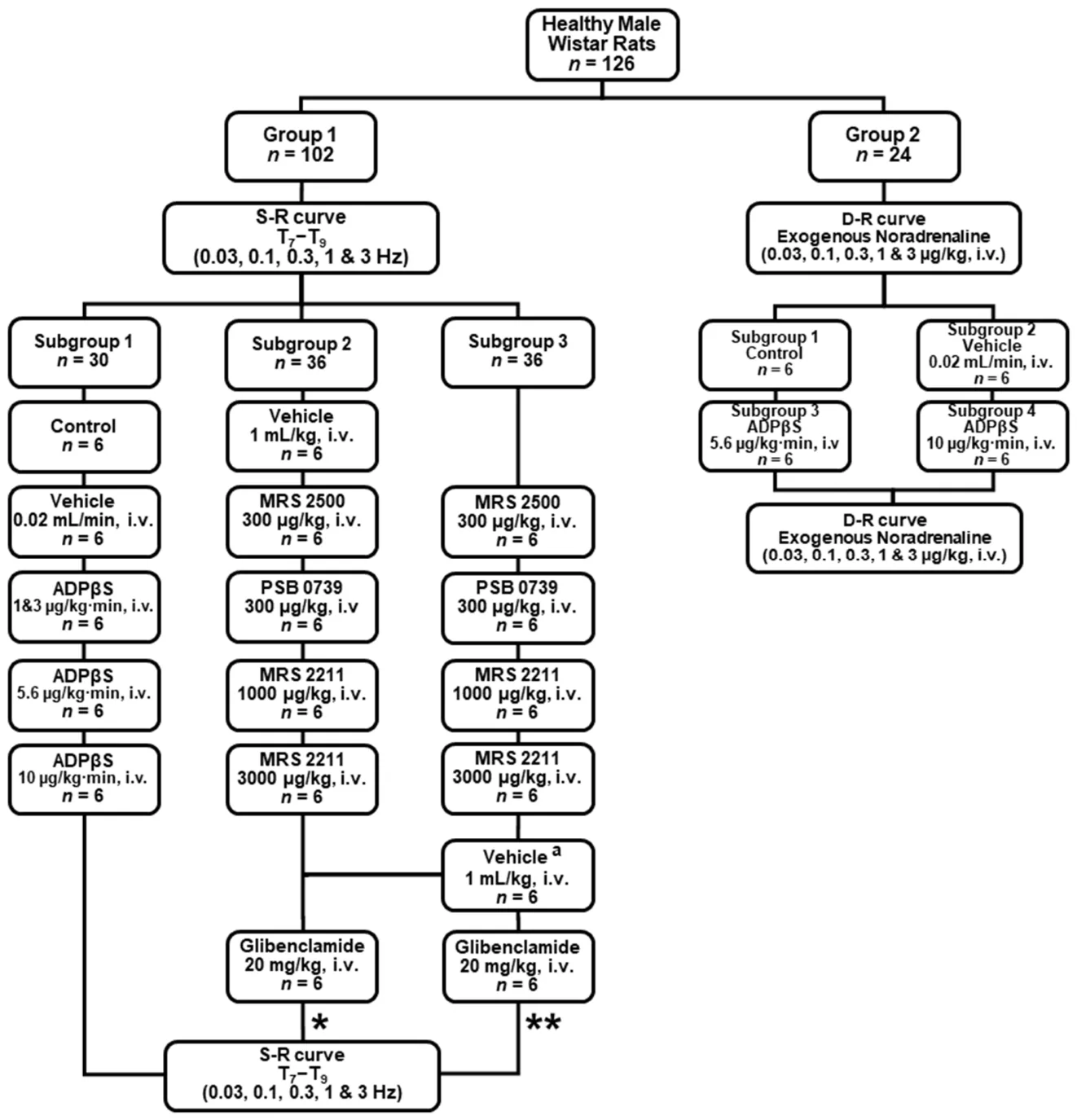
Experimental protocols. The total number of animals is shown, as well as their corresponding division into the two main groups and further subdivisions. In group 1 (S-R curves), all rats received gallamine (25 mg/kg, i.v.) before the start of the first S-R curve (for more details, see Section 4.3 Experimental Protocols). ^a^, Vehicle to dissolve glibenclamide (the vehicle mixture previously described; 1 mL/kg); *, Sympathetic responses elicited during the infusion of vehicle for ADPβS (bidistilled water; 0.02 mL/min, i.v.); **, Sympathetic responses elicited during the infusion of ADPβS (5.6 µg/kg·min, i.v.). S-R curve: Stimulus–Response curve; D-R curve: Dose–Response curve.

Once all animals (*n* = 126) achieved haemodynamic stability for at least 30 min, they were divided into two main groups (as depicted in Figure 7), namely, (i) group 1 (*n* = 102) for the investigation of the vasopressor responses produced by sympathetic electrical stimulation and (ii) group 2 (*n* = 24) for the analysis of the vasopressor responses produced by i.v. bolus injections of exogenous noradrenaline. The resultant S-R and D-R curves (produced by electrical stimulation and exogenous noradrenaline, respectively) were completed in approximately 35 min, with each electrical stimulus or dose administered every 3–7 min, as previously established [28,29,30,31,32,33].

#### 4.2.1. Group 1: Electrical Stimulation of the Vasopressor Sympathetic Tone

In accordance with the methods detailed for electrical stimulation of the rat vasopressor sympathetic tone [28,29,30,31,32,33], for the animals in group 1 (*n* = 102), the stainless steel rod left after pitching was replaced with an enamelled electrode, except for a 1 cm segment located 9 cm from the tip. Thus, the uncovered segment was placed in the T_7_–T_9_ region of the spinal cord to produce selective preganglionic stimulation of the vasopressor sympathetic tone [64]. Before electrical stimulation, this group was pretreated with an i.v. bolus of gallamine (25 mg/kg) to prevent electrically induced muscle spasms [28,29,30,31,32,33]. After a stabilization period of 30 min, diastolic blood pressure (a surrogate for peripheral vascular resistance [38]) and heart rate were measured and taken as the baseline values. “Then, the vasopressor sympathetic electrical stimulation was initiated by applying monophasic discharge trains of 10 s in duration, using rectangular pulses of 2 milliseconds at 50 volts, with increasing stimulation frequencies (i.e., 0.03, 0.1, 0.3, 1, and 3 Hz), as reported in previous studies [28,29,30,31,32,33]. Once blood pressure had returned to baseline values, the next stimulation frequency was applied until the S-R curve was completed”.

#### 4.2.2. Group 2: Intravenous Bolus Injections of Exogenous Noradrenaline

In the second group of rats (*n* = 24), as described earlier [28,29,30,31,32,33], no spinal electrode was used. Instead, the stainless steel pithing rod remained undisturbed in the vertebral canal throughout the experiment. After maintaining the rats in haemodynamically stable conditions for 30 min, baseline values of diastolic blood pressure and heart rate were determined. “Subsequently, i.v. boluses of exogenous noradrenaline (0.03, 0.1, 0.3, 1, and 3 μg/kg) were administered to produce dose-dependent vasopressor responses, as shown in previous studies [28,32]. Once diastolic blood pressure returned to baseline values, the next dose was administered until the D-R curve was completed”.

### 4.3. Experimental Protocols

As shown in Figure 7, (i) the animals in group 1 (*n* = 102) were partitioned into three distinct subgroups (*n* = 30, 36, and 36, respectively), and (ii) the animals in group 2 (*n* = 24) were classified into four smaller subgroups (*n* = 6 each). Then, the following four protocols were applied to these subgroups after completing the first S-R curve or D-R curve.

#### 4.3.1. Protocol 1: Evaluation of the Effect of a Continuous Infusion of Vehicle or ADPβS on the Vasopressor Responses Elicited by Sympathetic Electrical Stimulation

The first subgroup (*n* = 30) was further subdivided into five sets (*n* = 6 each), which received a continuous i.v. infusion of (i) nothing (control); (ii) vehicle (bidistilled water, 0.02 mL/min; twice); (iii) ADPβS (1 and 3 μg/kg·min); (iv) ADPβS (5.6 μg/kg·min); and (v) ADPβS (10 μg/kg·min). After 10 min, a second S-R curve was completed during the continuous i.v. infusion of the vehicle or the corresponding dose of ADPβS (see Figure 7). These infusion doses were selected based on our research group’s previous functional evidence on prejunctional purinergic neurovascular inhibition in pithed rats [24].

#### 4.3.2. Protocol 2: Analysis of the Direct Effect of the P2Y_1_ (MRS 2500), P2Y_12_ (PSB 0739) and P2Y_13_ (MRS 2211) Purinergic Receptor Antagonists, as Well as the K_ATP_ Channel Blocker (Glibenclamide) on the Sympathetic Vasopressor Responses

The second subgroup (*n* = 36) was classified into six sets (*n* = 6 each) receiving an i.v. bolus of (i) vehicle (bidistilled water, 1 mL/kg); (ii) MRS 2500 (300 μg/kg); (iii) PSB 0739 (300 μg/kg); (iv) MRS 2211 (1000 μg/kg); (v) MRS 2211 (3000 μg/kg); and (vi) glibenclamide (20 mg/kg). Ten minutes later, a continuous i.v. infusion of the vehicle (bidistilled water: 0.02 mL/min) was administered, and a second S-R curve was completed to determine the per se effect of the administered compounds (see Figure 7).

#### 4.3.3. Protocol 3: Investigation of the Effect of the P2Y_1_ (MRS 2500), P2Y_12_ (PSB 0739) and P2Y_13_ (MRS 2211) Purinergic Receptor Antagonists, as Well as the K_ATP_ Channel Blocker (Glibenclamide) on the Vascular Sympathoinhibition Induced by ADPβS

The third subgroup (*n* = 36) was segregated into six sets (*n* = 6 each) that were administered an i.v. bolus of (i) MRS 2500 (300 μg/kg); (ii) PSB 0739 (300 μg/kg); (iii) MRS 2211 (1000 μg/kg); (iv) MRS 2211 (3000 μg/kg); (v) the vehicle to dissolve glibenclamide (the vehicle mixture previously described; 1 mL/kg); and (vi) glibenclamide (20 mg/kg). Ten minutes later, all subgroups received a continuous i.v. infusion of ADPβS at a dose of 5.6 μg/kg·min. This was selected, based on the results of the first subgroup, as the infusion dose that produced the maximum sympathoinhibition, since a higher infusion dose produced no further sympathoinhibition (see Figure 2D,E). After 10 min, a second S-R curve was completed during the continuous i.v. infusion of ADPβS to evaluate the effects of the aforementioned compounds on the ADPβS-induced inhibition of the sympathetic vasopressor responses (see Figure 7). As previously demonstrated [21,23,24,65], the above antagonists or blockers were administered at supramaximal doses to ensure complete blockade of their target receptor-mediated responses in pithed rats.

#### 4.3.4. Protocol 4: Examination of the Effect of a Continuous Infusion of ADPβS on the Vasopressor Responses Elicited by Exogenous Noradrenaline

Once the D-R curve to exogenous noradrenaline (control curve) was completed, the second group of animals (*n* = 24) was divided into four subgroups (*n* = 6 each) receiving a continuous i.v. infusion of (i) nothing (control); (ii) vehicle (bidistilled water, 0.02 mL/min); (iii) ADPβS (5.6 μg/kg·min); and (iv) ADPβS (10 μg/kg·min). Ten minutes after the start of each infusion, a new D-R curve to noradrenaline was completed, as mentioned above, to observe the effect of the ADPβS infusion (Figure 7).

### 4.4. Compounds Used

In addition to the anaesthetic sodium pentobarbital (PISA Agropecuaria, Mexico City, Mexico), the compounds used in this study (purchased from the sources indicated) included gallamine triethyliodide, glibenclamide, D,L-noradrenaline hydrochloride, and adenosine 5′-O-(β-thio)-diphosphate trilithium salt (ADPβS) (Sigma Chemical Co., St. Louis, MO, USA); the tetraammonium salt of the dihydrogen phosphate ester of (1R*, 2S*)-4-[2-iodo-6-(methylamino)-9H-purin-9-yl]-2-(phosphonooxy)bicyclo [3.1.0]hexane-1-methanol (MRS 2500), the sodium salt of 1-amino-9,10-dihydro-9,10-dioxo-4-[[4-(phenylamino)-3-sulfophenyl]amino]-2-anthracenesulfonic acid (PSB 0739), and the sodium salt of 2-[(2-chloro-5-nitrophenyl)azo]-5-hydroxy-6-methyl-3-[(phosphonooxy)methyl]-4-pyridinecarboxaldehyde (MRS 2211) (Tocris Bioscience, Avonmouth, Bristol, UK).

As previously reported [28,29,30,31,32,33], “(i) gallamine and noradrenaline were dissolved in physiological saline; (ii) ADPβS, MRS 2500, PSB 0739, and MRS 2211 were dissolved in bidistilled water [21,23,24,54]; and (iii) glibenclamide was dissolved in a vehicle mixture consisting of 34% NaOH 0.2 M, 33% PEG, and 33% ethanol (1 mL/kg) [24,65]. In keeping with these studies, none of the aforementioned vehicles exerted significant effects (*p* > 0.05) on baseline diastolic blood pressure or heart rate values. All solutions of compounds were prepared immediately before each experiment and were administered i.v. at room temperature (approximately 23 °C). The doses used refer to the free base of the compounds, except for gallamine, whose dose refers to the corresponding salt”.

### 4.5. Supplementary Procedures

The doses of (i) bidistilled water (vehicle to dissolve ADPβS) and ADPβS were always given as continuous i.v. infusions at 0.02 mL/min by a KDS100 model infusion pump (KD Scientific Inc., Holliston, MA, USA), and (ii) antagonists/blockers and their corresponding vehicles were delivered via i.v. boluses at 1 mL/kg. To ensure that diastolic blood pressure returned to baseline values, intervals between subsequent stimulation frequencies or noradrenaline doses were adjusted according to the duration of individual vasopressor responses (range: 3–7 min; total recovery time: 7–10 min).

Furthermore, a distinctive advantage of the pithed rat model is that the vasopressor responses elicited by sympathetic electrical stimulation or exogenous noradrenaline are highly reproducible [28,29,30,31,32,33], which allowed us to analyse two or more S-R or D-R curves within the same animal (see experimental protocols and Figure 7). In contrast, the vasodepressor responses elicited by electrical stimulation of the neurovascular sensory CGRPergic tone or exogenous CGRP are typically not as reproducible [24,28,40,65,66].

### 4.6. Data Presentation and Statistical Analysis

All data shown in the graphs and text are expressed as mean ± standard error of the mean. To avoid overlapping and enhance visual clarity, error bars on figures containing multiple S-R or D-R curves are represented as one-sided intervals, alternating directions (upward or downward) between curves. As previously described [28,29,30,31,32,33], “the peak changes in diastolic blood pressure are presented as absolute values in mmHg (Δ Diast. blood pressure)”. Furthermore, to evaluate variations in diastolic blood pressure changes, a Student–Newman–Keuls test [67] was employed as a post hoc analysis after a two-way repeated-measures ANOVA confirmed significant overall differences. The threshold for statistical significance was established at *p* < 0.05.

Statistical processing was managed with SigmaPlot software (version 12.0, Systat Software Inc., San Jose, CA, USA), whereas graphical representations were prepared with GraphPad Prism software version 8.0.1 for Windows (GraphPad Software, San Diego, CA, USA, www.graphpad.com).

## 5. Conclusions

Our findings indicate that ADPβS (5.6 µg/kg·min) inhibits vasopressor sympathetic activity in pithed rats primarily by stimulating prejunctional P2Y_1_ receptors through K_ATP_ channel-dependent mechanisms. This prejunctional purinergic cascade highlights a novel homeostatic mechanism for modulating peripheral vascular resistance and identifies P2Y_1_ receptors as potential therapeutic targets for disorders characterised by sympathetic overactivity.

## Figures and Tables

**Table 1:** P2Y_1_, P2Y_12_, and P2Y_13_ purinergic receptor affinities for selected P2Y ligands.

	Receptors	P2Y_1_	P2Y_12_	P2Y_13_
Drugs				
**Agonist**	ADPβS	5.6	7.5	7.5
**Antagonists**	MRS 2500	**9.1**	4 ^a^	4 ^a^
	PSB 0739	6 ^b^	**7.6**	6 ^b^
	MRS 2211	5 ^c^	5 ^c^	**6.3**

Potency and affinity data for human receptors are reported as *p*IC_50_, *p*EC_50_, and *p*K_i_ values. Effects were only observed at concentrations of 100 μM, 1 μM, and 10 μM or higher for ^a^, ^b^, and ^c^, respectively. The numbers highlighted in red represent, by comparison, the highest affinity value for each antagonist. Data taken and adapted from Miguel-Martínez et al. [24].

## Data Availability

The original contributions presented in this study are included in the article. Further inquiries can be directed to the corresponding author.

## Abbreviations

ADP	Adenosine diphosphate
ADPβS	Adenosine 5′-O-(β-thio)-diphosphate
ATP	Adenosine triphosphate
CGRP	Calcitonin gene-related peptide
Diast. Blood Pressure	Diastolic blood pressure
D-R curve	Dose–Response curve
Glibencl.	Glibenclamide
S-R curve	Stimulus–Response curve

## References

[1] Burnstock G. (2017). Purinergic Signalling in the Cardiovascular System. Circ. Res..

[2] Burnstock G. (2017). Purinergic Signalling: Therapeutic Developments. Front. Pharmacol..

[3] Burnstock G., Ralevic V. (2014). Purinergic Signalling and Blood Vessels in Health and Disease. Pharmacol. Rev..

[4] Bautista-Pérez R., Franco M. (2024). Purinergic Receptor Antagonists: A Complementary Treatment for Hypertension. Pharmaceuticals.

[5] Silva-Velasco R.C., Haanes K.A., Villanueva-Castillo B., MaassenVanDenBrink A., Villalón C.M. (2026). Pharmacological Profile of Purinergic P2Y Receptors Involved in Neurogenic Cardiovascular Modulation Induced by ADP and Related Compounds in Rats. J. Integr. Neurosci..

[6] von Kügelgen I., Nörenberg W., Koch H., Meyer A., Illes P., Starke K. (1999). P2-Receptors Controlling Neurotransmitter Release from Postganglionic Sympathetic Neurones. Prog. Brain Res..

[7] Abbracchio M.P., Burnstock G., Boeynaems J.M., Barnard E.A., Boyer J.L., Kennedy C., Knight G.E., Fumagalli M., Gachet C., Jacobson K.A. (2006). International Union of Pharmacology LVIII: Update on the P2Y G Protein-Coupled Nucleotide Receptors: From Molecular Mechanisms and Pathophysiology to Therapy. Pharmacol. Rev..

[8] Zimmermann H. (2021). History of Ectonucleotidases and Their Role in Purinergic Signaling. Biochem. Pharmacol..

[9] Erb L., Weisman G.A. (2012). Coupling of P2Y Receptors to G Proteins and Other Signalling Pathways. Wiley Interdiscip. Rev. Membr. Transp. Signal..

[10] IJzerman A.P., Jacobson K.A., Müller C.E., Cronstein B.N., Cunha R.A. (2022). International Union of Basic and Clinical Pharmacology. CXII: Adenosine Receptors: A Further Update. Pharmacol. Rev..

[11] Lazarowski E.R. (2012). Vesicular and Conductive Mechanisms of Nucleotide Release. Purinergic Signal..

[12] von Kügelgen I. (2006). Pharmacological Profiles of Cloned Mammalian P2Y-Receptor Subtypes. Pharmacol. Ther..

[13] von Kügelgen I. (2019). Pharmacology of P2Y Receptors. Brain Res. Bull..

[14] von Kügelgen I. (2024). Pharmacological Characterization of P2Y Receptor Subtypes—An Update. Purinergic Signal..

[15] Mahdi A., Jiao T., Tratsiakovich Y., Yang J., Östenson C.G., Pernow J., Zhou Z. (2018). Altered Purinergic Receptor Sensitivity in Type 2 Diabetes-Associated Endothelial Dysfunction and Up_4_A-Mediated Vascular Contraction. Int. J. Mol. Sci..

[16] Bogdanov Y.D., Dale L., King B.F., Whittock N., Burnstock G. (1997). Early Expression of a Novel Nucleotide Receptor in the Neural Plate of *Xenopus* Embryos. J. Biol. Chem..

[17] Kauffenstein G., Tamareille S., Prunier F., Roy C., Ayer A., Toutain B., Loufrani L., Henrion D. (2016). Central Role of P2Y_6_ UDP Receptor in Arteriolar Myogenic Tone. Arterioscler. Thromb. Vasc. Biol..

[18] Abbracchio M.P., Burnstock G. (1994). Purinoceptors: Are There Families of P2X and P2Y Purinoceptors?. Pharmacol. Ther..

[19] Nishimura A., Sunggip C., Oda S., Numaga-Tomita T., Tsuda M., Nishida M. (2017). Purinergic P2Y Receptors: Molecular Diversity and Implications for Treatment of Cardiovascular Diseases. Pharmacol. Ther..

[20] Rodríguez-Rodríguez R., Yarova P., Winter P., Dora K.A. (2009). Desensitization of Endothelial P2Y_1_ Receptors by PKC-Dependent Mechanisms in Pressurized Rat Small Mesenteric Arteries. Br. J. Pharmacol..

[21] Silva-Velasco R.C., Villanueva-Castillo B., Haanes K.A., MaassenVanDenBrink A., Villalón C.M. (2023). Pharmacological Nature of the Purinergic P2Y Receptor Subtypes That Participate in the Blood Pressure Changes Produced by ADPβS in Rats. Pharmaceuticals.

[22] Quintas C., Fraga S., Gonçalves J., Queiroz G. (2009). The P2Y_1_ and P2Y_12_ Receptors Mediate Autoinhibition of Transmitter Release in Sympathetic Innervated Tissues. Neurochem. Int..

[23] Villanueva-Castillo B., Rivera-Mancilla E., Haanes K.A., MaassenVanDenBrink A., Villalón C.M. (2020). The Role of Purinergic P2Y_12_ and P2Y_13_ Receptors in ADPβS-Induced Inhibition of the Cardioaccelerator Sympathetic Drive in Pithed Rats. Purinergic Signal..

[24] Miguel-Martínez A.D., Linares-Bedolla J., Villanueva-Castillo B., Haanes K.A., MaassenVanDenBrink A., Villalón C.M. (2023). Pharmacological Profile of the Purinergic P2Y Receptors That Modulate, in Response to ADPβS, the Vasodepressor Sensory CGRPergic Outflow in Pithed Rats. Pharmaceuticals.

[25] Gray D.W., Marshall I. (1992). Human α-Calcitonin Gene-Related Peptide Stimulates Adenylate Cyclase and Guanylate Cyclase and Relaxes Rat Thoracic Aorta by Releasing Nitric Oxide. Br. J. Pharmacol..

[26] Hong K.W., Yoo S.E., Yu S.S., Lee J.Y., Rhim B.Y. (1996). Pharmacological Coupling and Functional Role for CGRP Receptors in the Vasodilation of Rat Pial Arterioles. Am. J. Physiol..

[27] Smillie S.J., King R., Kodji X., Outzen E., Pozsgai G., Fernandes E., Marshall N., de Winter P., Heads R.J., Dessapt-Baradez C. (2014). An Ongoing Role of α-Calcitonin Gene-Related Peptide as Part of a Protective Network against Hypertension, Vascular Hypertrophy, and Oxidative Stress. Hypertension.

[28] Avilés-Rosas V.H., Rivera-Mancilla E., Marichal-Cancino B.A., Manrique-Maldonado G., Altamirano-Espinoza A.H., MaassenVanDenBrink A., Villalón C.M. (2017). Olcegepant Blocks Neurogenic and Non-Neurogenic CGRPergic Vasodepressor Responses and Facilitates Noradrenergic Vasopressor Responses in Pithed Rats. Br. J. Pharmacol..

[29] Villalón C.M., Contreras J., Ramírez-San Juan E., Castillo C., Perusquía M., López-Muñoz F.J., Terrón J.A. (1995). 5-Hydroxytryptamine Inhibits Pressor Responses to Preganglionic Sympathetic Nerve Stimulation in Pithed Rats. Life Sci..

[30] Villalón C.M., Contreras J., Ramírez-San Juan E., Castillo C., Perusquía M., Terrón J.A. (1995). Characterization of Prejunctional 5-HT Receptors Mediating Inhibition of Sympathetic Vasopressor Responses in the Pithed Rat. Br. J. Pharmacol..

[31] Villalón C.M., Centurión D., Rabelo G., de Vries P., Saxena P.R., Sánchez-López A. (1998). The 5-HT_1_-like Receptors Mediating Inhibition of Sympathetic Vasopressor Outflow in the Pithed Rat: Operational Correlation with the 5-HT_1A_, 5-HT_1B_ and 5-HT_1D_ Subtypes. Br. J. Pharmacol..

[32] Manrique-Maldonado G., González-Hernández A., Marichal-Cancino B.A., Villamil-Hernández M.T., Del Mercado O.A., Centurión D., Villalón C.M. (2011). The Dopamine Receptors Mediating Inhibition of the Sympathetic Vasopressor Outflow in Pithed Rats: Pharmacological Correlation with the D_2_-like Type. Basic Clin. Pharmacol. Toxicol..

[33] Ruiz-Salinas I., González-Hernández A., Manrique-Maldonado G., Marichal-Cancino B.A., Altamirano-Espinoza A.H., Villalón C.M. (2013). Predominant Role of the Dopamine D_3_ Receptor Subtype for Mediating the Quinpirole-Induced Inhibition of the Vasopressor Sympathetic Outflow in Pithed Rats. Naunyn-Schmiedeberg’s Arch. Pharmacol..

[34] Kotecha N., Coffa F.P. (1999). NO Mediates Postjunctional Inhibitory Effect of Neurogenic ACh in Guinea Pig Small Intestinal Microcirculation. Am. J. Physiol. Heart Circ. Physiol..

[35] Pohl U., Busse R. (1990). Endothelium-Dependent Modulation of Vascular Tone and Platelet Function. Eur. Heart J..

[36] Tykocki N.R., Boerman E.M., Jackson W.F. (2017). Smooth Muscle Ion Channels and Regulation of Vascular Tone in Resistance Arteries and Arterioles. Compr. Physiol..

[37] North R.A. (2002). Molecular Physiology of P2X Receptors. Physiol. Rev..

[38] Lefferts W.K., Lefferts E.C., Hibner B.A., Fernhall B., Chirinos J.A. (2022). Role of the Heart and Arterial Tree in Physiologic Adjustments during Exercise. Textbook of Arterial Stiffness and Pulsatile Hemodynamics in Health and Disease.

[39] Altamirano-Espinoza A.H., González-Hernández A., Manrique-Maldonado G., Marichal-Cancino B.A., Ruiz-Salinas I., Villalón C.M. (2013). The Role of Dopamine D_2_, but Not D_3_ or D_4_, Receptor Subtypes, in Quinpirole-Induced Inhibition of the Cardioaccelerator Sympathetic Outflow in Pithed Rats. Br. J. Pharmacol..

[40] Taguchi T., Kawasaki H., Imamura T., Takasaki K. (1992). Endogenous Calcitonin Gene-Related Peptide Mediates Nonadrenergic Noncholinergic Depressor Response to Spinal Cord Stimulation in the Pithed Rat. Circ. Res..

[41] Marichal-Cancino B.A., Manrique-Maldonado G., Altamirano-Espinoza A.H., Ruiz-Salinas I., González-Hernández A., MaassenVanDenBrink A., Villalón C.M. (2013). Analysis of Anandamide- and Lysophosphatidylinositol-Induced Inhibition of the Vasopressor Responses Produced by Sympathetic Stimulation or Noradrenaline in Pithed Rats. Eur. J. Pharmacol..

[42] Villamil-Hernández M.T., Alcántara-Vázquez O., Sánchez-López A., Centurión D. (2013). The α_2_-Adrenoceptors Mediating Inhibition of the Vasopressor Sympathetic Outflow in Pithed Rats: Pharmacological Correlation with α_2A_, α_2B_ and α_2C_ Subtypes. Eur. J. Pharmacol..

[43] Kenakin T. (2004). Principles: Receptor Theory in Pharmacology. Trends Pharmacol. Sci..

[44] Brayden J.E. (2002). Functional Roles of K_ATP_ Channels in Vascular Smooth Muscle. Clin. Exp. Pharmacol. Physiol..

[45] Dogan M.F., Yildiz O., Arslan S.O., Ulusoy K.G. (2019). Potassium Channels in Vascular Smooth Muscle: A Pathophysiological and Pharmacological Perspective. Fundam. Clin. Pharmacol..

[46] Quayle J.M., Nelson M.T., Standen N.B. (1997). ATP-Sensitive and Inwardly Rectifying Potassium Channels in Smooth Muscle. Physiol. Rev..

[47] Burnstock G. (2020). Introduction to Purinergic Signalling. Methods in Molecular Biology.

[48] Malmsjö M., Erlinge D., Högestätt E.D., Zygmunt P.M. (1999). Endothelial P2Y Receptors Induce Hyperpolarisation of Vascular Smooth Muscle by Release of Endothelium-Derived Hyperpolarising Factor. Eur. J. Pharmacol..

[49] Schicker K.W., Chandaka G.K., Geier P., Kubista H., Boehm S. (2010). P2Y_1_ Receptors Mediate an Activation of Neuronal Calcium-Dependent K^+^ Channels. J. Physiol..

[50] Oe K., Sperlágh B., Sántha E., Matkó I., Nagashima H., Foldes F.F., Vizi E.S. (1999). Modulation of Norepinephrine Release by ATP-Dependent K^+^-Channel Activators and Inhibitors in Guinea-Pig and Human Isolated Right Atrium. Cardiovasc. Res..

[51] Goto K., Fujii K., Abe I., Fujishima M. (2000). Sympathetic Control of Arterial Membrane Potential by ATP-Sensitive K^+^-Channels. Hypertension.

[52] Thomas G.D., Hansen J., Victor R.G. (1997). ATP-Sensitive Potassium Channels Mediate Contraction-Induced Attenuation of Sympathetic Vasoconstriction in Rat Skeletal Muscle. J. Clin. Investig..

[53] Jackson W.F. (2005). Potassium Channels in the Peripheral Microcirculation. Microcirculation.

[54] Haanes K.A., Labastida-Ramírez A., Blixt F.W., Rubio-Beltrán E., Dirven C.M., Danser A.H.J., Edvinsson L., MaassenVanDenBrink A. (2019). Exploration of Purinergic Receptors as Potential Anti-Migraine Targets Using Established Pre-Clinical Migraine Models. Cephalalgia.

[55] Centurión D., Huerta de la Cruz S., Gutiérrez-Lara E.J., Beltrán-Ornelas J.H., Sánchez-López A. (2016). Pharmacological Evidence That NaHS Inhibits the Vasopressor Responses Induced by Stimulation of the Preganglionic Sympathetic Outflow in Pithed Rats. Eur. J. Pharmacol..

[56] Al Shoyaib A., Archie S.R., Karamyan V.T. (2020). Intraperitoneal Route of Drug Administration: Should It Be Used in Experimental Animal Studies?. Pharm. Res..

[57] Grassi G., Quarti-Trevano F., Cuspidi C., Sanidas E., Mancia G., Thomopoulos C. (2025). Sympathetic Overactivation in the Resistant Hypertensive Phenotype: A Meta-Analysis of Published Studies. Hypertension.

[58] Villalón C.M., Centurión D., del Mar Fernández M., Morán A., Sánchez-López A. (1999). 5-Hydroxytryptamine Inhibits the Tachycardia Induced by Selective Preganglionic Sympathetic Stimulation in Pithed Rats. Life Sci..

[59] (1999). Especificaciones Técnicas para la Producción, Cuidado y Uso de los Animales de Laboratorio.

[60] Bayne K. (1996). Revised Guide for the Care and Use of Laboratory Animals Available. Physiologist.

[61] McGrath J.C., Drummond G.B., McLachlan E.M., Kilkenny C., Wainwright C.L. (2010). Guidelines for Reporting Experiments Involving Animals: The ARRIVE Guidelines. Br. J. Pharmacol..

[62] Kleinman L.I., Radford E.P. (1964). Ventilation Standards for Small Mammals. J. Appl. Physiol..

[63] Shipley R.E., Tilden J.H. (1947). A Pithed Rat Preparation Suitable for Assaying Pressor Substances. Proc. Soc. Exp. Biol. Med..

[64] Gillespie J.S., MacLaren A., Pollock D. (1970). A Method of Stimulating Different Segments of the Autonomic Outflow from the Spinal Column to Various Organs in the Pithed Cat and Rat. Br. J. Pharmacol..

[65] Cuesta C., García-Pedraza J.Á., García M., Villalón C.M., Morán A. (2014). Role of 5-HT_7_ Receptors in the Inhibition of the Vasodepressor Sensory CGRPergic Outflow in Pithed Rats. Vasc. Pharmacol..

[66] Villalón C.M., Albarrán-Juárez J.A., Lozano-Cuenca J., Pertz H.H., Görnemann T., Centurión D. (2008). Pharmacological Profile of the Clonidine-Induced Inhibition of Vasodepressor Sensory Outflow in Pithed Rats: Correlation with α_2A/2C_-Adrenoceptors. Br. J. Pharmacol..

[67] Steel R.G.D., Torrie J.H. (1980). Principles and Procedures of Statistics: A Biometrical Approach.

